# Theories and Management of Aging: Modern and Ayurveda Perspectives

**DOI:** 10.1093/ecam/nep005

**Published:** 2011-06-08

**Authors:** Hema Sharma Datta, S. K. Mitra, Rangesh Paramesh, Bhushan Patwardhan

**Affiliations:** ^1^Interdisciplinary School of Health Sciences, University of Pune, Pune-411007, India; ^2^Himalaya Health Care, Research & Development, Makali, Bangalore 562 123, India

## Abstract

Aging is a complex phenomenon, a sum total of changes that occur in a living organism with the passage of time and lead to decreasing ability to survive stress, increasing functional impairment and growing probability of death. There are many theories of aging and skin remains the largest organ of the study. Skin aging is described as a consequence of intrinsic and extrinsic factors. The most common amongst visible signs of skin aging are wrinkles and there are various therapies including antiaging cosmeceuticals, sunscreens, chemical peeling, injectable agents, such as botox, fibrel, autologous fat grafting as also few surgical procedures have been used. Ayurveda, the Indian traditional medicine, describes aging with great details. This review provides modern and Ayurvedic perspectives on theories and management of aging.

## 1. Introduction

Aging is a universal process that probably began with the origin of life. Accumulation of the diverse deleterious changes produced by aging throughout the cells and tissues progressively impairs function and can eventually cause death. Aging changes can be attributed to development, genetic defects, the environment, disease and an inborn process—the aging process [1]. The aging of the world's population has profound implications for medical care and health care systems. According to the United Nations, the number of people worldwide aged 60 years or older will increase from 1 in 10 currently to 1 in 5 by 2050. By 2050, the ratio of people aged 65 years or older to those aged 15–64 years will double in developed nations and triple in developing nations. This demographic shift compels us to confront the changes associated with aging and the various antiaging therapies [2].

## 2. Theories of Aging

### 2.1. Modern Perspective

Aging is complex phenomena generally defined by gerontologists as a process that results in an age-related increase of death rate or failure rate. Biologists define aging as the sum total of all changes that occur in a living organism with the passage of time and lead to a decreasing ability to survive stress, functional impairment and death [3]. The most common theories include Mutation Accumulation and Antagonistic Pleiotropy theory, the two theories posit that aging is due to pleiotropic genes with beneficial early-life effects but deleterious late-life effects (antagonistic pleiotropy) or mutations with purely deleterious late-life effects (mutation accumulation) [4].

Reliability theory consists of a body of ideas, mathematical models and methods directed to predict, estimate, understand and optimize the life span distribution of systems and their components [5]. Programmatic Theory states that aging is a preordained process due to an inherent genetic program, played out at a rate characteristic of each species, this theory takes in consideration aging genes, cellular senescence, telomere shortening, failure of apoptosis and longevity genes [6]. Stochastic Theory, which states that random cumulative environmental damage to genes and proteins produces aging and homeostatic failure, takes in consideration oxidative stress (free radical damage), amino acid racemization and nonenzymatic glycolysation [7]. Other theories, such as Random Chemical Damage and Information Transfer theories [8] are based around the idea of genetic damage and impaired information transfer. Double-Agent theory is a new, unifying synthesis which argues that there is a tradeoffs between oxidative stress as a critical redo signal that marshals genetic defenses against physiological stress and oxidative stress as a cause of aging [9].

### 2.2. Ayurvedic Perspective

Aging is known as “*Jarā*" defined as that which has become old by the act of wearing out “*jīryati iti jarā*”. It is synonymed as “*vārdhakya*” meaning increasing age [10]. Ayurveda divides human life into—childhood (up to the age 16 years); youth and middle age [from 16 to 60 years (c*haraka*) or 70 years (*sushruta*) and exhibits progressively the traits of growth (*vivardhamana*, 16–20 years of age), youth (*youvana*, 20–30 years), maturity (*sampoornata*, 30–40 years), deterioration (*parihani*, 40 years onwards) which gradually sets in up to 60 years]; old age, wherein after 60–70 years the body elements, sense organs, strength, and so forth. begin to decay [11].

While describing aging, *Ayurveda* takes in consideration *Prana* (life energy that performs respiration, oxygenation and circulation). It governs two other subtle essence *ojas* and *tejas*. *Ojas* (the essence of the seven *dhatus* or bodily tissues) is responsible for the auto-immune system and mental intelligence, it is necessary for longevity. Displaced *ojas* creates the *kapha*-related disorders and decreased *ojas* creates *vata*-related reactions. *Tejas* (the essence of a very subtle fire or energy) governs metabolism through the enzyme system. *Agni* (central fire or energy source in the body) promotes digestion, absorption and assimilation of food. *Tejas* is necessary for the nourishing and transformation of each *dhatu*. Aggravated *tejas*, burns away *ojas* reducing immunity and overstimulating *pranic* activity. Aggravated *prana* produces degenerative disorders in the *dhatus*. Lack of *tejas* results in over production of unhealthy tissue and obstructs the flow of *pranic* energy. Just as it is essential to maintain balance amongst the *tridosha*—*vata, pitta, kapha* principles of motion, metabolism, structure, respectively, the *dhatus* and the three *malas* (bodily wastes); it is also important for longevity that *prana*, *ojas* and *tejas* remain in balance. The *tridosha* play a very important role in the maintenance of cellular health and longevity. *Kapha* maintains longevity on the cellular level. *Pitta* governs digestion and nutrition. *Vata*, which is closely related to pranic life energy, governs all life functions. Proper diet, exercise and lifestyle can create a balance among these three subtle essences, ensuring long life [12].

## 3. Skin Aging

Skin aging is a complex process determined by the genetic endowment of the individual and the environmental factors [13]. The most obvious signs of aging skin are atrophy, laxity, wrinkling, sagging, dryness, a multiplicity of pigmented other blemishes and sparse gray hair [14]. Symptoms of chronological aging include dry and thin skin, fine wrinkles, abnormal blood vessels, age spots, benign and malignant skin tumors due to the deterioration of the skins immune system. Intrinsic skin aging is determined primarily by genetic factors and hormonal status. Photo aging is a separate process and largely involves damage to the collagen and the elastin fibers in the skin. The deleterious effects of solar radiation on dermal connective tissue leads to visible manifestations of photo aging termed as premature aging. The UVB rays directly interact with the DNA of the cutaneous cells where as the deleterious effects of UVA are principally due to the formation of free radical oxygen, which result in an alteration in the nuclear and also mitochondrial DNA and also an activation of enzymes, metalloprotenase, capable of damaging the extra cellular matrix [15, 16].

In Ayurveda, *Charaka* has described *twak* (skin) in six layers, he has named the first two as *udakadara (bahyatwak)* and *astrikdhara* and has not named the remaining four layers. *Sushruta* has described the same in seven layers viz. *avabhasini*, *lohita, shweta, tamra, vedini, rohini* and *mamsadhara*. *Avabhasini*, the outermost layer, reflects the complexion and the quality of the *Rasa Dhatu* (nutrient fluid, the first of the seven tissues of the body). It also acts as a mirror as it indicates whether the physiology as a whole is balanced or imbalanced, and whether there is inner health or disorder; it also reflects the aura of the individual. *Mamsadhara* is the innermost layer is the platform for the skin's stability and firmness. When this layer is in balance, the skin looks young and supple. A skin product that has a *vayasthapana* (antiaging) effect nourishes this layer to help retard the aging process. *Vata* skin which is dry, thin, fine pored, delicate and cool to touch tends to develop wrinkles earlier than the other skin types. *Pitta* skin which is fair, soft, warm and medium thickness is photosensitive and has least tolerance to sun and is most likely to accumulate sun damage over the years. *Kapha* skin which is thick, oily, soft and cool to touch tends to develop wrinkles much later in life than *Vata* or *Pitta* type but because of its thickness and oiliness, is more prone to accumulate *ama* (toxins) under the skin [17, 18].

## 4. Wrinkles

Wrinkles are result of dermal-hypodermal junction and shrinking of the superficial muscles, which have their points of insertion at the dermi. With reduction in muscle mass, skin thickness, diminished elasticity of dermal collagen and elastin and drying of the stratum corneum, the resulting behavioral change observed in the skin is loss of mechanical strength and viscoelasticity. Development of fine wrinkles begins to take place at the age of 30s, reaching a peak in 40s but tending to rather decrease from the 60s and over, deep wrinkles are considered to be increasing in the 50s. Wrinkles are formed and promoted by both internal and external factors. Internal factors include aging, changes in the endocrine system and nerve system and hereditary factors. External factors include exposure to UV rays and the oxidation or drying associated with UV exposure [19].

## 5. Antiaging Approaches

The human skin loses its antioxidation ability with age, by exposure to the surrounding environment for a considerable period of time, and the skin undergoes emaciation as a result of the formation of peroxylipids. Wrinkles are considered to appear as an outcome of this emaciation. Hence use of products, which enhance antioxidation, should be taken into consideration. Even brief exposure to UV radiation increases the activity of enzymes that break down the proteins collagen and elastin that provide structural support for the skin, thus pretreatment of skin with creams containing actives that can reduce the activation of these enzymes is recommended. Use of sunscreens, which minimize the harmful effects of solar radiation is also advisable [20–22].

## 6. Antiaging Therapies

### 6.1. Topical Agents

Various topical application products that delay and/or reverse visible signs of aging are termed as antiaging cosmeceuticals; the active ingredients that are most commonly used in such products include Vitamins A, C and E, hyaluronic acid, alpha hydroxy acids (AHAs), beta hydroxy acids (BHAs) and essential fatty acids (EFAs). Retinoids are used in topical treatment of photo-aged skin, as it appears to increase the rate of cell division and improves wrinkling, coarseness, hyper pigmentation and roughness associated with over exposure to the sun. It repairs photoaged skin by inhibiting collagenase and improving dermal vasculature, while also stimulating new collagen deposition, it promotes the down growth of rate ridges, restoring the undulating dermo-epidermal interphase and improves the skin's water barrier properties [23]. Hyaluronic acid helps to keep the surface of the skin hydrated, supple and less prone to wrinkling; when used in cosmeceutical products it forms an undetectable transparent film. Restylane (hyaluronic acid), an injectable gel that acts as a filler to remove the wrinkle is used which binds to the water and provides volume to fill in larger folds of skin [24]. AHAs, BHAs can cause increased skin thickness, improvement in skin elasticity and increased collagen content and glycosaminoglycans. AHAs (ascorbic acid, citric acid, gluconic acid, glycolic acid, lactic acid, malic acid, mandelic acid and tartaric acid) and the BHAs (salicylic acid and pyruvic acid) are used in cosmetic products [25]. Vitamin C can accelerate wound healing; it is a potent antioxidant that protects fatty tissues from oxidation damage and play an integral role in elastin and collagen synthesis. It is capable of controlling inflammatory responses associated with UV exposure. Vitamin E has significant moisturizing properties, anti-inflammatory effects and may provide protection from UV damages. It acts as an antioxidant and inhibits the formation of lipid peroxides and thus prevents skin aging. It is known to improve decreased function of the sebaceous gland and ameliorate excessive pigmentation in the skin [26]. Other topical antioxidants include leaf wax of *Eucalyptus* and *Prunus*, the seeds of black rice, leaves of barley, sesame seeds, rosemary green tea, turmeric, beta-carotene, coral extracts, aloe, and so forth. Natural antioxidants include flavonoids (anthocyanins; red grapes, blueberries, strawberries, red cabbage), quercetin (onions, apple skins, berries, broccoli), catechins (green tea, cacao), isoflavones (soybeans), carotenoids (carrots, sweet peppers, oranges), lycopene (tomatoes), oligomeric proanthocyanidins or procyanidins (grape seed extract). EFAs are building blocks of cellular membranes which allow efficient transportation of nutrients from the extracellular space into the intracellular environment where metabolism takes place. Rosehip seed oil (Rosa Mosqueta) which is high in EFAs smoothes wrinkles by hydrating the skin and slows new signs of aging. Estrogen topical application shows improvement in skin elasticity and firmness, increase in skin moisture and collagen synthesis and decrease in wrinkle depth. Lipoic acid is a potent scavenger with anti-inflammatory properties, it has beneficial effects on photo-aged skin [27, 28].

Sunscreens are of two types: chemical sunblocks, such as PABA, PABA esters, benzophenones, salicylates and anthranilates contain molecules that mainly absorb the UVB radiation, while physical sunblocks, such as titanium dioxide, magnesium silicate, zinc oxide, red petrolatum and kaolin place a coating on the skin that reflects the light. UVA exposure can produce elastic tissue damage, actinic skin damage and contribute to the formation of skin cancer. The only sunscreen agents that can completely block both the UVB and UVA wavelengths are physical or opaque sunblocks [29]. Chemical peeling is a safe and efficient treatment for moderate facial skin aging; in this treatment, chemicals remove layers of skin which results in smoother texture less evident fine wrinkles and evident lightening of hyperpigmentations. Commonly used peeling agent is glycolic acid and pyruvic acid [30].

### 6.2. Injectable Agents

Botox is a neuromuscular blocking agent produced by *Clostridium botulinum*. Botulinum toxin type A, when injected in hyperactive corrugator superciliaries or procerus muscles of the face that controls frowning, it produces a transient localized muscle weakness resulting in temporary improvement in frown lines. The application of injectable chemodenervation with botulinum toxin type A has become a useful and significant tool for facial rejuvenation [31]. Bovine collagen implant is used for cosmetic purposes, principally on the face to diminish wrinkles, such as glabellar creases and prominent nasolabial folds. Indeed, correction with all forms of bovine collagen appears to be lost as the material is displaced in the human from its site of implantation in the dermis into the subcutaneous space [32]. Fibrel is composed of porcine gelatin powder and epsilon—amino caproic acid that is reconstituted with the patient's plasma and 0.9% sodium chloride. Fibrel works by creating a clot that becomes colonized by host collagen. This technique has shown good long-term persistent results of correction (2–5 years) in treated individuals [33]. Autologous fat grafting is a standard method for soft tissue augmentation; used for volume restoration of the aging phase in which following liposuction the harvested fat is reinjected immediately. Fat is the ideal filler because it is natural, nonallergenic and readily available [34].

### 6.3. Surgical Procedures

Cutaneous surgery is performed to treat visible signs of aging in extremely elderly patients and is well tolerated ensuring comfort and safety even in the oldest patients [35]. Blepharoplasty or eyelid surgery is a minimally invasive procedure that can be accomplished with a small incision and can restore a youthful appearance to the aging face. Laser-assisted blepharoplasty is a further simplified technique [36]. Dermabrasion surgically abrades or planes the epidermis and dermis with the use of a rapidly rotating wire brush or diamond fraise followed by wound healing which allows re-epithelialization to occur from the underlying adnexal structures. During the maturation phase of wound healing, fibroblasts replace and remodel collagen bundles in the papillary and upper reticular dermis and results in smoothing out fine wrinkles [37].

### 6.4. Ayurveda and Skin Health

According to Ayurveda, a number of factors determine skin health and youthfulness, and these include proper moisture balance (*Kapha* in balance), effective functioning of the metabolic mechanisms that coordinate all the various chemical and hormonal reactions of the skin (*Pitta* in balance), and efficient circulation of blood and nutrients to the different layers of the skin (*Vata* in balance). The health of the following three types of body tissue are especially reflected in the skin: nutritional fluid (Rasa), blood (*Rakta*) and muscle (*Mamsa*). To be effective, an antiaging application has to provide support to all of these areas.

Antiaging treatment includes two types of therapies *Urjaskara* (promotive) and *Vyadhihara* (curative). For *vata* skin to stay youthful skin care products that can nourish and rehydrate the skin should be used otherwise it may be susceptible to wrinkles and premature aging. Warm oil self-massage and all natural moisturizers may help. For *pitta* skin good sunscreens for protection from the sun, good facial skin oils should be used daily. Tanning treatments and therapies that expose delicate sensitive skin for extended periods of time to steam/heat should be avoided. For *kapha* skin a daily warm oil massage and cleansing of skin with gentle exfoliant should be done.

### 6.5. Rejuvenation Therapy

Ayurveda describes several processes to address control and prevention of aging. *Pancha Karma* is one of the popular rejuvenation and detoxification process that consists of three stages including *Purva Karma* (pretreatment), *Pradhana Karma* (primary treatment) and *Paschat Karma* (posttreatment). *Snehana* (oleation) and *Swedana* (sudation) are the two *Purva Karma* procedures. The four *Pradhan Karma* include *Vamana* (medical emesis), *Virechana* (purgation), *Nasya* (nasal administration), *Basti* (enema). A school of thought from *Sushruta* also considers *Raktamokshana* (bloodletting) as one of the *Pancha Karma*. *Paschat Karma* (posttreatment) mainly deals with *Ahar* (diet) regimens, *Vihar* (exercise) and use of health-promoting *Rasayana* and other medicines.

There have been few studies indicating physiological benefits to *Panchakarma*. Ayurveda describes various rejuvenative therapies with help of special class of medicinal preparations called *Rasayana* that are believed to rebuild the body, mind, prevent degeneration and postpone aging or rather reverse the aging process. *Charaka* has described two methods of rejuvenation, the first method—intramural (*kutipravesika*) required the subject to remain inside a chamber in isolation and second method which was less rigorous and was carried out in open air—extramural (*vatatapika*). The intramural method is suitable for healthy, self-controlled, wise, strong and affluent persons whereas extramural method is advisable for others. In intramural method, a special cottage is constructed on an auspicious land facing east or north, it is safe and supplied with all the necessary articles for treatment and the procedure is started on an auspicious day. In extramural therapy, its basically the use of various medicinal plant formulations [38–42]. According to *Ayurveda*, the practice of *yoga*, which is a disciplined science of life, is a very important, natural, preventive measure to ensure good health [43, 44].

## 7. Conclusion

While there are various theories and approaches to management of aging, the traditional knowledge remains important both in understanding the process and effective management. Several interventions have been tried for treatments of various conditions primarily arising as a result of aging. Since aging process has been experienced by human beings for several generations, the traditional knowledge from various parts of the world provide easy, natural and holistic ways for healthy aging. Ayurveda, the great Indian tradition also offers conceptual framework on various theories and concepts of aging process. Ayurveda also offers time tested therapies for healthy aging. With the vast information available in Ayurvedic literature on aging and skin care, one can explore the possibilities of developing new antiaging or antiwrinkle treatments with the natural ingredients for topical applications. Few pharmaceutical companies have used Ayurvedic knowledge for developing skin toning, fairness creams or sun tan lotions. There are antiwrinkle formulations available in market containing *Shorea robusta* resins. However, more systematic scientific studies to establish the safety and efficacy of Ayurvedic therapies in both rejuvenation and detoxification procedures are needed. Thus, this review may be interesting to modern scientists to understand different approaches and processes that may be useful to progress research on promotive, preventive and therapeutic interventions generally on various degenerating conditions and aging.
